# Serological Parameters and Vascular Investigation for a Better Assessment in DVT during Pregnancy—A Systematic Review

**DOI:** 10.3390/medicina57020160

**Published:** 2021-02-10

**Authors:** Catalina Filip, Demetra Gabriela Socolov, Elena Albu, Cristiana Filip, Roxana Serban, Radu Florin Popa

**Affiliations:** 1 Saint Spiridon University Hospital, Vascular Surgery Clinic, Independence Boulevard no. 1, 700111 Iasi, Romania; catalina.filip@umfiasi.ro; 2 Department of Obstetrics and Gynecology, University of Medicine and Pharmacy “Grigore T. Popa”, 700111 Iasi, Romania; 3 Department of Pharmacology, University of Medicine and Pharmacy “Grigore T. Popa”, 700111 Iasi, Romania; 4 Department of Biochemistry, University of Medicine and Pharmacy “Grigore T. Popa”, 700111 Iasi, Romania; roxana.serban@umfiasi.ro; 5 Department of Vascular Surgery, University of Medicine and Pharmacy “Grigore T. Popa”, 700111 Iasi, Romania; radu.popa@umfiasi.ro

**Keywords:** venous thromboembolism, pulmonary embolism, anticoagulants, thrombophilia

## Abstract

Pregnancy and the postpartum period represent a condition characterized by a thrombotic predisposition. The majority of pregnant women do not face acute or severe thrombotic events. In general, mild inconveniences such as leg swelling or moderately painful thrombotic events (phlebitis) are encountered. However, when pregnancy is associated with inherited or acquired deficits that affect homeostasis, the risk of acute or even life-threatening events can increase significantly. The major consequence is the loss of the fetus or the venous thromboembolism that endangers the mother’s life. Venous thromboembolism is caused by deep vein thrombosis, therefore timely detection and especially the assessment of the extent of the thrombotic event are crucial. In this paper we have summarized the most important paraclinical investigations. The study emphasizes the importance of selecting the methods of investigation. The right choice allows establishing a correct diagnosis and individualizing the treatment.

## 1. Introduction

Deep vein thrombosis (DVT) is an important medical problem, especially for women of childbearing potential. Deep vein thrombosis means the formation of a blood clot in the deep veins, especially in the lower limbs and only occasionally in the upper limbs.

The symptoms of the disease are swelling of the affected limb accompanied by pain, and redness and heat in the affected area, but almost half of the cases have no symptoms [1].

Severe complications of the disease are pulmonary embolism and post-thrombotic syndrome. The occurrence of DVT can be idiopathic or as a result of risk factors such as contraceptives, obesity, surgical maneuvers, trauma, and so forth.

Venous thrombosis can be occlusive (acute) and non-occlusive (less symptomatic/asymptomatic) or chronic if it is symptomatic and persists for more than 10 days [2].

Regarding the affected area, DVT is classified as proximal (ilio-femoral) when it is located above the knee and distal (in the leg) when it is located below the knee.

In most cases DVT develops in the leg or thigh of the popliteal or ilio-femoral vein and evolves in the direction of venous flow to the heart and can reach the inferior vena cava [3].

An important aspect of DVT is a different pathophysiology compared to arterial thrombosis. Arterial thrombosis occurs as a result of a vascular wall injury in an environment with a normal oxygen content. The arterial clot contains a significant percentage of platelets. In contrast, DVT may occur in the absence of vascular damage in a low-oxygen environment. The venous clot contains mainly erythrocytes and fibrin but also white blood cells and platelets. The presence of white blood cells, which play an important role in the adhesion of cells to the surface of the endothelium, indicates an inflammatory phenomenon in the formation of clot [4]. Hypoxemia, increased by venous stasis, activates certain cellular mechanisms that induce the association of monocytes with endothelial proteins, thus promoting clot formation [5].

In pregnancy, the deep vein thrombosis is characterized by the Virchow triad: venous stasis, hypercoagulability, and endothelial damage. Deep vein thrombosis is not a frequent complication but is a very serious medical problem. While in underdeveloped countries mortality at birth is caused by hemorrhage, in developed countries mortality is caused by the thromboembolic complications [1].

During pregnancy, DVT can be caused by prolonged rest/immobilization or obesity. Other responsible factors are preeclampsia, varicose veins, multiple pregnancies, and hereditary or acquired thrombophilia.

The frequency of DVT is similar over the three trimesters of pregnancy but increased in the first six weeks postpartum [1]. Deep vein thrombosis associated with pulmonary thromboembolism is known as venous thromboembolism. In pregnancy the risk of venous thromboembolism is 5 times higher [6], and for the case of associated hereditary, thrombophilia is up to 30 times higher [1,7].

Nowadays the first pregnancy currently occurs in older ages. In many cases, the age for the first pregnancy has moved to 35–45 years, so the number of pregnancies obtained through fertilization has constantly increased. We can say that these aspects characterize a so-called “contemporary pregnancy”. Therefore, the contemporary pregnancy characterized by old age and/or assisted reproduction presents the following thrombotic risks: hyperstimulation that generates high levels of estradiol, which promotes hemoconcentration and immobilization, which favors hypercoagulability, which promotes DVT.

Regarding the location of the injured area and the moment of occurrence of DVT, literature [3] indicates some particularities of DVT as follows:

- in more than 88% of cases, it affects the left lower limb as a result of the compression on the left common iliac vein accentuated by the growth of the uterus [6]
- it occurs most commonly in the third pregnancy trimester on the ilio-femoral vein
- localization above the groin is much more common compared with other non-pregnant DVT patients
- it frequently occurs, postpartum, in the first six weeks after birth

## 2. The Research Was Performed as Follows

The literature search was done on Pub Med, ScienceDirect, Google Scholar, DOAJ, UpToDate in the last 20 years.

Literature selection was made based on keywords: venous thromboembolism, pulmonary embolism, anticoagulants, thrombophilia.

The inclusion criteria covered the following aspects: reviews on basic pathophysiology, clinical evaluation in DVT, serological parameters currently used in the evaluation of DVT, serological parameters useful in the assessment of DVT envisaged for implementation, anticoagulant therapy.

The exclusion criteria were: normal non-DVT pregnancy, thrombosis and venous stroke, upper extremity thrombosis, surgical therapy of DVT during pregnancy, DVT pregnancy associated with non-thrombophilia pathology.

The selected articles were used as shown in Table 1 [1,2,3,4,5,6,7,8,9,10,11,12,13,14,15,16,17,18,19,20,21,22,23,24,25,26,27,28,29,30,31,32,33,34,35,36,37,38,39,40,41].

## 3. Epidemiology

Normal pregnancy and the postpartum period represent a condition characterized by a thrombotic predisposition. The hypercoagulable physiological state of pregnancy and postpartum seems to prevent blood loss during pregnancy and excessive bleeding at birth. For this reason, pregnant women develop DVT five times more frequently than non-pregnant women [1]. For patients with no thrombotic event in history, the overall incidence of DVT in pregnancy is up to 2/1000 [8]. During normal pregnancy, the frequency of thrombosis is similar in all three trimesters. The frequency of thrombotic event is much higher in pregnancy with associated risk factor, such as: inherited or acquired thrombophilia, history of thrombosis, antiphospholipid syndrome, lupus [1]. Other independent risk factors are age 35 and older, zero parity, multiple pregnancies, obesity and immobility, which increase the risk by 1.5–2 times [5]. Assisted reproduction is considered a risk factor, as it increases up to 10 times compared to natural pregnancy [1,6]. Finally, the postpartum period (six weeks) has an increased risk of DVT with an incidence up to 10 times higher compared to natural pregnancy [1].

## 4. Laboratory Assessment of DVT

It is well known that normal pregnancy is associated with a hypercoagulation state generated by the modification of the serum concentration of specific parameters. Thus, coagulation factor II, VII, X, prothrombin, fibrinogen, and its degradation products (D-dimer) are increased. On the contrary, the fibrinolytic activity of the specific proteins involved in the process, such as protein C and protein S, is decreased. This hypercoagulation state promotes thrombosis, but in normal pregnancy there are isolated acute events. If in the pregnant state additional factors that promote coagulation are added, the risk of a severe thrombotic event suddenly increases. Risk factors that promote DVT include: history of thrombotic event, thrombophilia (antithrombin deficiencies, antiphospholipid antibodies, Leiden factor V, methylenetetrahydrofolate reductase (MTHFR), etc.), use of contraceptives, and prolonged immobilization. Particular situations such as older age and assisted reproduction can also be risk factors for a DVT occurring event. The literature specifies that, in the previously presented situations, the risk of DVT increases 1.5–2 times [6,7].

For patients with no thrombotic event in history, the overall incidence of DVT in pregnancy and the puerperium is up to 2/1000 [8], thus supplementary tests for thrombophilia are not consider mandatary. On the contrary, laboratory investigation of thrombophilia is compulsory for the following pregnant patient categories: family history of DVT events in first-degree relatives [9,10], patients with previous different thrombotic event, suspicion of antiphospholipid syndrome. During pregnancy, for all pregnant women with thrombophilia and for those in older age or in assisted reproduction pregnancies, the detection of DVT risk as early as possible prevents acute events such as miscarriage and allows the identification of patients who have contraindications to antithrombotic therapy.

Therefore, for a reliable assessment of a possible deep vein thrombosis event, two aspects must be followed: firstly, the evaluation of the coagulation/fibrinolysis parameters, and secondly, the determination of the extent of the contribution of the particular risk factors such as thrombophilia.

To estimate the level of risk of DVT during pregnancy, laboratory tests should follow a few steps to maximize the accuracy of the diagnosis.

A first step refers to the medical history of the patient that can highlight any medical event that may be associated with a disturbance of the coagulation–fibrinolysis balance. The medical history of the patients must be as complete as possible and must contain information about a possible antithrombotic therapy.

The second step regards the routine coagulation tests that include: fibrinogen, prothrombin time, thrombin time, activated partial thromboplastin time, fibrinogen and D-dimers. The purpose of this first group of analyses is to determine whether the patient has one or more of the risk factors that can trigger a DVT event [11]. In the case of a positive test, the third step is the determination of the individual thrombophilia risk factors previously identified. The method/methods used for the individual risk factor determination must be selected according to the specificity and sensitivity of the method. The acquired data will be correlated to assess the extent of the DVT risk. A reliable assessment will facilitate the decision to choose the appropriate therapy to be followed.

For the evaluation of the coagulation/fibrinolysis balance in DVT, the following are currently determined: prothrombin time (PT), activated partial thromboplastin time (APTT), fibrinogen and D-dimers.

Currently for the diagnosis of DVT, the determination of D-dimers is considered the most reliable diagnosis tool in non-pregnant patients [12]. D-dimers are produced after fibrinogen degradation under plasmin activity. They are generated when coagulation cascade is activated. Regarding the determination of D-dimers during pregnancy, numerous studies [13,14] indicate different reference values of D-dimers depending on the trimester of the pregnancy. Thus, plasma concentration of D-dimers increases progressively during pregnancy [15] with poor predictive value in excluding the diagnosis of deep vein thrombosis. Although there is some consensus in this regard, some authors believe that the risk of thromboembolism can be ruled out with certainty if D-dimer values are negative and compression duplex ultrasonography is normal and thus, the test presents high efficacy for the negative prediction of DVT rather than the positive one [16,17].

During labor, D-dimers usually increase significantly, after which they decrease rapidly at three days postpartum and slowly return to normal values four weeks later. From this moment on, the test regains its usefulness. However, recent studies show that the onset of postpartum hemorrhage is preceded by hyperfibrinolysis. If D-dimers do not return to normal or their concentration increases, then postpartum hemorrhage should be considered [18].

If, after routine determination of the coagulation–fibrinolysis balance, one or more individual risk factors are identified, the laboratory investigation of the individual thrombophilia will begin.

In cases of inherited or acquired thrombophilia, the classification of the risk of DVT as small, moderate, or severe is of clinical importance [19,20,21] because it allows the individualization of therapy. As not all thrombophilia investigations are affordable or routine tests, the selection of the pregnant patients to be tested should be considered. A first strategy is that once a risk factor is identified, all known risk factors should be investigated.

**Figure 1:**
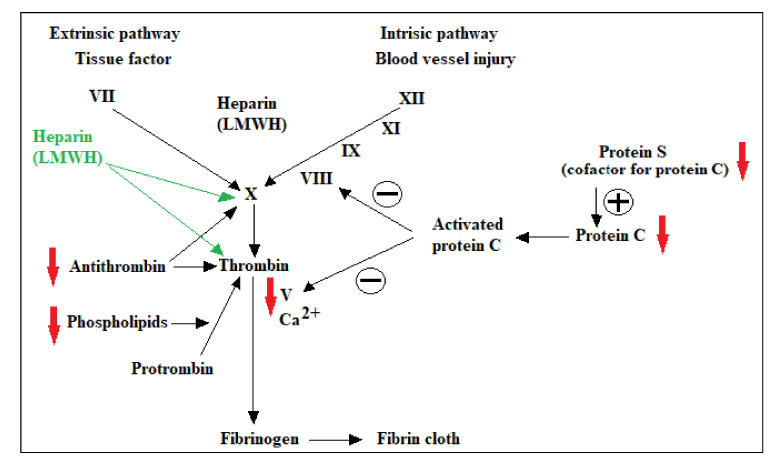
Specific proteins of the coagulation cascade that can become risk factors in acquired or inherited deficiencies, thus promoting hypercoagulation. Targeted factors for antithrombotic therapy.

Laboratory investigations of thrombophilia include the following individual risk factors: activated protein C resistance and V Leiden factor, prothrombin G20210A mutation, antithrombin deficiency, proteins C and S deficiencies, antiphospholipid antibodies, increased factor VIII level, as shown in Figure 1, and hyperhomocysteinemia. In order to exclude false-positive results, specific confirmations are requested, as follows: mandatory repeating testing for antiphospholipid antibodies, increased factor VIII level, hyperhomocysteinemia or measurement of specific antigen for antithrombin deficiency, proteins C and S deficiencies.

For the inherited thrombophilia caused by deficiencies of activated protein C resistance and factor V Leiden or prothrombin G20210A mutation, literature shows that the associated risk of developing DVT is considered low for heterozygosity and highest for homozygosity [22]. Thus, the most appropriate method of investigation is supposed to be DNA analysis. Because the DNA test is not routine, the first diagnosis is established based on the functional test for the activated protein C resistance and factor V Leiden. If a positive result occurs, then the DNA analysis is performed.

The inherited thrombophilia may occur in the deficiency of three individual risk factors: antithrombin, protein C, and protein S. From the clinical point of view, these parameters are considered as being very reliable markers for a moderate risk of DVT [22]. The protein deficiencies come from two different causes: a low synthesis that generates insufficient quantities of the protein or a low quality of the synthesized protein that generates poor activity. As a consequence, two ways of investigation are available to determine these deficiencies: a functional test that assesses the activity and the immunoassay test that assesses the quantity of these proteins. As a general rule, both tests are performed in order to avoid a false-positive test. The deficiencies of all these tree proteins are rare, but among them antithrombin and protein C are considered a preponderant hereditary thrombophilia risk factor.

Another thrombophilia risk factor is the presence of antiphospholipid antibodies that trigger the antiphospholipid syndrome. Antiphospholipid syndrome is considered one of the important thrombophilia risk factors. It is involved in both arterial and venous thromboembolism and in miscarriage as well. In patients with a history of recurrent pregnancy loss, old age, and assisted reproduction, the earlier assessment of antiphospholipid antibodies is recommended in order to establish and to start the appropriate therapy. To avoid false-positive results, the laboratory determination should be performed twice at a distance of about 12 weeks.

Hyperhomocysteinemia (HHC) is another thrombophilia risk factor. HHC is characterized by increased concentration of homocysteine (HC) in plasma, and is associated with both venous and arterial thrombosis [23,24,25,26]. It is generally accepted that HHC disrupts normal endothelial functions, which are the relaxation of blood vessels and the state of anticoagulation of the blood, thus disturbing coagulation–fibrinolysis balance [27,28]. Hyperhomocysteinemia is caused by an inherited or acquired deficiency of the enzymes involved in its metabolism (methylenetetrahydrofolate reductase or cystathionine beta synthetase) or a deficiency of vitamins belonging to the group of B vitamins.

Based only on serological routine factors and on additional individual risk factor, Dargaud et al. [29] proposed a Lyon DVT score in order to assess as accurately as possible the risk of the DVT event. Lyon DVT score indicates the severity and the initiation of the therapy as follows: <3 there is no antenatal prophylaxis; 3–5 LMWH starting on the third trimester; ≥6 LMWH starting during the first trimester (VTE, venous thromboembolism; DVT, deep venous thrombosis; LMWH, low-molecular-weight heparin) [29].

## 5. Clinical Vascular Evaluation of DVT

During pregnancy, a large number of women experience vascular events that are generally moderate such as superficial varicose veins, or more important ones such as compression of the abdominal vessels as a result of the evolution of pregnancy. Acute events that may also occur, although less frequent, are of major importance and life threatening. Acute thrombotic events mainly include venous thromboembolism and post-thrombotic syndrome. The DVT investigation from the vascular point of view includes the same steps that can not only improve the diagnosis but also detect earlier a hidden pathology.

From the vascular point of view, DVT detection can be accomplished by the following steps:

- the examination of the lower limbs should be performed on both the whole limb and on segments, for patients with suspicion/risk of DVT (according to current literature recommendations [30].
- examination by ultrasonography as the first line of investigation in DVT due to lack of risks. It is less sensitive than venography but it is an affordable and available tool. Even so, it has low accuracy in pelvic vein thrombosis. Although ultrasonography is the main DVT method of determination, magnetic resonance imaging (MRI) is recommended when ultrasound is inconclusive and the clinical symptoms persist. A high-quality study [31] shows that the prevalence of venography over ultrasonography in DVT identification in asymptomatic patients is about 22%. Even so, the study concluded that particularly for the proximal veins, ultrasound is accurate in diagnosing DVT in asymptomatic patients having a 95% confidence. For the distal vein, the ultrasound accuracy in DVT detection is inconclusive due to anatomical particularities.
- examination of venography only where other investigations are inconclusive. Venography is generally accepted as the gold standard in detecting DVT. Its limitation comes from the invasive method of investigation, and thus non-invasive diagnostic tests like ultrasound replace venography in routine screening for DVT, particularly in pregnant patients. In conclusion, the ultrasound investigation is the gold standard in particular cases of pregnancy.

Comprehensive information on the tools used by clinicians to detect and assess the extent of a thrombotic event, including DVT, in patients of all categories, including coronavirus disease-2019 patients, can be found in the literature [32,33,34,35].

**Figure 2:**
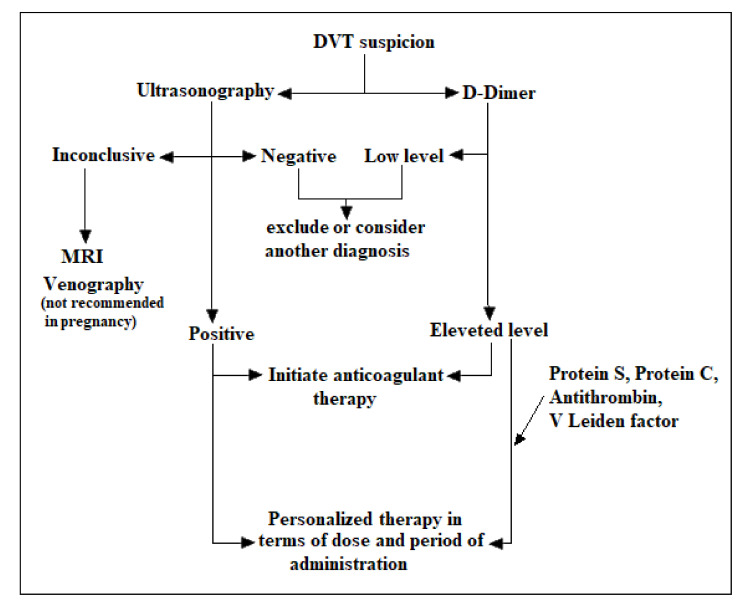
Clinical and serological investigation of deep vein thrombosis (DVT) in pregnancy.

In conclusion, a general summary for the clinical DVT investigation in pregnancy is presented in Figure 2.

## 6. Diagnosis and Therapeutic Steps

The diagnosis of DVT is generally made on clinical grounds. The most common symptom of DVT is swelling and discomfort in the lower extremities in the absence of trauma as shown in Table 2. DVT can be present in normal and complicated pregnancies as well as in the postpartum period.

The clinical diagnosis is accompanied by laboratory tests that, in this case, aim to highlight particular situations such as thrombophilia.

D-dimer represents the choice analysis of screening in thrombophilia and has a high negative predictive value. However, D-dimer levels increase during normal or complicated pregnancy as well as in the postpartum period, which makes this parameter less reliable. For this reason, the investigation of fibrinolytic-specific parameters, presented in Table 3, appears to be mandatary particularly in thrombophilia.

## 7. Therapeutic Steps during Pregnancy

Anticoagulation with heparin is the therapy of choice in DVT. However, the choice of treatment must take into account certain particular factors/steps:

Before initiating treatment, laboratory tests must establish the thrombophilia state. Although a positive result does not significantly influence the therapy, the type of thrombophilia may influence the doses and duration of anticoagulants.

DVT treatment must prevent not only the thromboembolism but also prevent PTS?

The renal condition is decisive in choosing the type of anticoagulant.

Thrombolytic therapy with plasminogen activators (tPA, urokinase, and streptokinase) is relatively contraindicated in pregnancy because of the theoretical risk of massive abruption.

## 8. Antithrombotic Treatment

International experts from the National Institute of Health Care and Excellence (NICE) established the general guidelines for antithrombotic therapy including pregnant patients [36,37]. The guidelines contain indication for deep vein thrombosis as well as pulmonary embolism (PE).

## 9. General Guidelines in DVT and PE

In case of suspicion of DVT or PE, the optimal moment for the early initiation of anticoagulant treatment will be decided, so that it can continue even after the diagnosis is confirmed.

In March 2020, direct-acting anticoagulants and some low-molecular-weight heparins (LMWH) were considered off-label for indication in the treatment of suspected cases of DVT or PE.

Before initiating the anticoagulant therapy, biological tests must be performed (complete blood count, renal and hepatic function, prothrombin time, activated partial thromboplastin time) and anticoagulation must be initiated until results are received and doses adjusted accordingly within the next 24 h.

In confirmed cases of DVT or PE, anticoagulant therapy is performed for three months. Comorbidities (obesity, hemodynamic instability, neoplasms, renal failure, antiphospholipid syndrome) are taken into account when initiating anticoagulant treatment.

Either apixaban or rivaroxaban should be indicated in patients with proximal DVT or confirmed PE. If they are not available, LMWH will be administered for at least five days followed by dabigatran or edoxaban or LMWH simultaneously with a vitamin K antagonist (VKA) for at least five days or until the international normalized ratio (INR) is at least 2.0 in two consecutive readings, followed by VKA monotherapy. Unfractionated heparin (UFH) is usually not associated with a VKA for the proximal DVT or confirmed PE therapy, except in cases of renal impairment or an increased risk of bleeding.

In patients with proximal DVT or PE both confirmed and with renal impairment (creatinine clearance estimated between 15 mL/min and 50 mL/min), one of the following will be chosen: (a) apixaban, rivaroxaban, LMWH for at least five days, followed by edoxaban or (b) dabigatran if the estimated creatinine clearance is 30 mL/min or greater, (c) LMWH or UFH, co-administered with a VKA for at least five days or until the INR is at least 2.0 in two consecutive determinations, followed by a VKA monotherapy.

In patients with proximal DVT or PE both confirmed with pre-existing renal impairment (estimated creatinine clearance less than 15 mL/min), choose one of the following: (a) LMWH, UFH, LMWH or (b) UFH simultaneously with VKA for at least five days or until the INR is at least 2.0 in two consecutive determinations, followed by a VKA alone.

Long-term anticoagulation for secondary prevention will be decided after assessing the benefits and risks that may occur from continuing, stopping, or changing the anticoagulant [38,39,40].

Catheter thrombolytic therapy will be considered in patients with DVT with symptoms lasting less than 14 days, good functional status (ECOG 0-1), life expectancy of more than one year, and low risk of bleeding.

Pharmacological systemic thrombolytic therapy is recommended in patients with hemodynamic instability PE.

In patients with proximal DVT or in whom anticoagulation is contraindicated or in whom an episode of PE occurred during anticoagulant treatment, a filter in the inferior vena cava is indicated.

## 10. General Guidelines in Thrombophilia before and after Childbirth

As for therapy in pregnancy, the guideline indicates the use of heparin (small molecule) as it can reduce the risk of obstetric adverse events.

In the particular case of thrombophilia (inherited or acquired), the use of low doses of low-molecular-weight aspirin may or may not be associated with heparin during the pregnancy [41].

When a single thrombotic risk factor is present, prophylaxis with LMWH heparin is recommended both before and after childbirth. When two thrombotic risk factors are present, LMWH heparin prophylaxis is recommended starting from the 28th week of pregnancy and continuing after birth.

As a general rule, LMWH heparin prophylaxis for the postnatal period recommends a general period of six weeks of administration.

## 11. Conclusions

Pregnancy, thrombophilia, deep vein thrombosis, and thromboembolism are causally linked. The diagnosis of DVT is made on clinical symptoms and D-dimers, after which a general scheme of anticoagulant treatment is applied. Thus, the presence of a hidden thrombophilia risk may be missed. Therefore, the chosen therapy may be excessive or not fully efficient. Although there are recommendations for the detection, evaluation, and treatment of thrombophilia during pregnancy, the ultimate goal is to select the appropriate investigations to make the best possible therapeutic decision. Our study focused on the importance of selecting investigational tools and highlighted relevant serological factors that, when included in routine analyses of pregnancy tests, allow treatment to be personalized.

## Figures and Tables

**Table 1:** The targeted main issues.

Key Summary	References
Clinical vascular assessment	[2,30,31,33,34,35]
Serological assessment	[8,9,11,13,14,16,17,18,20,23,24,25,26]
Management and therapy in DVT-pregnancy	[6,22,27,28,29,36,39,40]
Review and protocols	[1,3,4,5,7,10,12,15,19,21,32,37,38,41]

**Table 2:** Clinical Investigation in DVT diagnosis.

Symptoms and Signs of Deep Venous Thrombosis
Leg swelling
Leg pain or discomfort and walking difficulty
Increased temperature and edema
Tenderness
Lower abdominal pain (sometimes associated)

**Table 3:** Blood/serum investigation in DVT diagnosis.

Blood and Serum Modification in Deep Venous Thrombosis
Elevated white cell count
Prothrombin time (PT) and a partial thromboplastin time (PTT)
Homocysteine levels (to detect inherited or acquired deficiencies)
Factor V Leiden and prothrombin (factor II) 20210 mutation (to detect inherited risk factors)
Protein C and protein S (to detect inherited risk factors or deficiency in blood clotting factors)
Lupus anticoagulant testing (to diagnose antiphospholipid syndrome)

## Data Availability

The study did not report any data.
